# Reducing infantile anemia: insight on patterns of process and outcome indicators by ethnicity and socioeconomic class during a 10-year intervention program and 5 years after

**DOI:** 10.1186/s13584-021-00510-9

**Published:** 2022-01-05

**Authors:** Joseph Meyerovitch, Doron Carmi, Shraga Aviner, Michael Sherf, Doron Comaneshter, Yoseph Laks, Calanit Key, Uri Gabbay, Arnon D. Cohen

**Affiliations:** 1 Medicine Wing, Community Division, Clalit Health Services, Tel Aviv, Israel; 2 The Jesse Z and Sara Lea Shafer Institute for Endocrinology and Diabetes, National Center for Childhood Diabetes, Schneider Children’s Medical Center of Israel, Petach Tikva, Israel; 3 Sackler Faculty of Medicine, Tel Aviv University, 6997801 Ramat Aviv, Tel-Aviv, Israel; 4 Shoham Ambulatory Center, Clalit Health Services, Shoham, Israel; 5 Department of Pediatrics, Faculty of Health Sciences, Barzilai University Medical Center, Ashkelon, Israel; 6 Faculty of Health Sciences, Ben-Gurion University of the Negev, Beer Sheba, Israel; 7 Clalit Health Services, Tel Aviv, Israel; 8 Pediatric Ambulatory Center, Clalit Health Services, Tel Aviv, Israel; 9 Nursing Medicine Wing, Community Division, Clalit Health Services, Tel Aviv, Israel; 10 Quality Unit, Rabin Medical Center, Petach Tikva, Israel; 11 Siaal Research Center for Family Medicine and Primary Care Faculty of Health Sciences, Ben Gurion University of Negev, Beer Sheva, Israel

**Keywords:** Infantile anemia (IA), Intervention program, Process, Outcome, Yield

## Abstract

**Background:**

In 2005, Clalit Health Services (CHS), the largest health maintenance organization in Israel, initiated an intervention program aimed at reducing the prevalence rate of infantile anemia (IA). This study evaluated the progress made during the intervention (2005–2014) and its yield 5 years after it ended (2019).

**Methods:**

The CHS database was retrospectively reviewed twice yearly from 2005 to 2014 for repetitive samples of children aged 9 to 18 months regarding the previous half-year interval, and a single sample in 2019. Data were collected on gender, ethnicity (Jewish/non-Jewish), socioeconomic class (SEC; low/intermediate/high), hemoglobin testing (yes/no), and hemoglobin level (if tested). Excluded were infants with documented or suspected hemoglobinopathy.

**Results:**

At study initiation, the rate of performance of hemoglobin testing was 54.7%, and the IA prevalence rate was 7.8%. The performance rate was lower in the Jewish than the non-Jewish subpopulation. The low-SEC subpopulation had a similar hemoglobin testing rate to the high-SEC subpopulation but double the IA prevalence rate. Overall, by the end of the intervention (2014), the performance rate increased to 87.5%, and the AI prevalence rate decreased to 3.4%. In 2019, there was little change in the performance rate from the end of the intervention (88%) and the IA prevalence was further reduced to 2.7%. The non-Jewish and low-SEC subpopulations showed the most improvement which was maintained and even bettered 5 years after the intervention ended.

**Conclusions:**

The 10-year IA intervention program introduced by CHS in 2005 led to a reduction in IA prevalence rate to about 3.5% in all sub-populations evaluated. By program end, the results in the weaker subpopulations, which had the highest prevalence of IA at baseline, were not inferior to those in the stronger subpopulations. We recommended to the Israel Ministry of Health to adopt the intervention countrywide, and we challenge other countries to consider similar interventions.

## Introduction

Anemia currently affects 24.8% of the world’s population, with the highest prevalence in the preschool age group. The most significant cause is iron deficiency. In 2002, iron-deficiency anemia (IDA) was included among the important contributing factors to the global burden of disease [1]. The mean reported global prevalence rate of IDA in children aged 0.5–4.99 years is 47.4% [2], ranging from 21% in Europe to 67.6% in Africa. Studies from the United States reported IDA rates of 8% to 14% in children aged 1 to 3 years [3–5].

The rapid growth characteristic of children in their first year of life places them at risk of infantile IDA (IIDA) owing to their increasing need for iron and a lack of dietary iron sources. Factors exacerbating this risk include maternal anemia during pregnancy, prematurity, low birth weight, introduction of cow's milk or exclusive breastfeeding after 6 months, low socioeconomic class (SEC), and birth to newly immigrated parents [6–8]. IIDA has been found to be related to slow growth [1, 2, 9], impaired immune response [9], poor development [9, 10], and intellectual and behavioral problems, all of which appear to last into adulthood [9, 11]. As dietary counseling during the first year of life has proved ineffective, iron supplement remedies are crucial [12]. Implementation of national programs for the prevention of IIDA has improved the health of both infants and the general public and reduced the financial burden on healthcare systems [9, 13].

Clalit Health Services (CHS) is the largest health maintenance organization in Israel, with nearly 5 million members, providing countrywide primary pediatric and many other health services. A previous CHS-based population study by our group showed that 15.5% of infants in Israel had anemia [14]. The prevalence was high in the non-Jewish relative to the Jewish subpopulation (22.5% vs. 10.5%) and in low SECs [14]. Accordingly, in 2005, CHS initiated an active intervention program designed to address this issue. Oral presentations were included in clinical staff meetings explaining the importance of preventing infantile anemia (IA) in general, and of conducting complete blood count tests and prescribing iron remedies to anemic infants in particular. Physicians were made aware of recent evidence-based studies, and the nursing staff was educated about the consequences of IA. In addition, leaflets in three languages (Hebrew, Arabic, Russian) on preventing IA were distributed to parents. To gauge the effect of the program, CHS defined two quality indicators: the incidence of infants who underwent hemoglobin testing (performance indicator), and the prevalence rate of anemia (outcome indicator). The quantified aim of each indicator was "a moving target", and both were updated with the progress of the intervention. Primary pediatricians received quarterly feedback reports on compliance with the performance and prevalence targets together with a list of children aged 9–18 months who required a hemoglobin test. A notification was also attached to the electronic medical record of these children to alert their primary physicians at the next visit. In addition, physicians periodically received a list of children found to have low hemoglobin as a reminder to initiate or continue iron treatment.

The predefined aims of the program were reached in 2014, when the program was terminated. Monitoring of the two quality indicators continues to date.

The aim of the present study was to evaluate the progress of the intervention in terms of the two quality indicators and the yield at the end of the intervention and also 5 years later. Moreover, we sought to determine if the program equally benefited weaker and stronger subpopulations.

## Methods

### Data source

CHS manages a computerized data warehouse that captures clinical, administrative and financial information from its network of hospitals, clinics, pharmacies, and laboratories and can be accessed down to the individual patient. The data warehouse is linked to national databases providing socio-demographic data.

### Study design and data collection

The CHS database was retrospectively retrieved twice yearly from 2005 to 2014 for repetitive samples of children aged 9 to 18 months regarding the previous half-year interval, and as a single sample at the end of 2019.

Data were collected on gender, ethnicity (Jewish/non-Jewish), SEC (low/intermediate/high), hemoglobin testing (yes/no), and hemoglobin level (if tested). Excluded were infants with a documented or suspected hemoglobinopathy, such as thalassemia (by mean corpuscular volume), impaired hemoglobin F, or hemoglobin A2, such that IA could be used as a proxy for IIDA.

### Evaluation

For each half-year interval, the hemoglobin-testing performance rate was calculated for all eligible infants in the respective sampled population. The performance rate pattern described the rate of infants who underwent at least one hemoglobin test in consecutive half-year intervals. The IA prevalence rate was calculated only for those tested and designated as undetermined for the remainder. The outcome pattern described the prevalence rate of infants with anemia in the consecutive half-year intervals. Progress of the program was determined by the pattern of hemoglobin-testing performance rate and pattern of IA prevalence rate over time. The overall effectiveness of the program was determined by the outcome at the end of the intervention and the yield 5 years later.

### Definitions

IA was defined as hemoglobin < 105 gr/L, as in our earlier cross-sectional study using the same database and same aged children [14].

For infants who underwent more than one hemoglobin test in a given half-year interval, the highest hemoglobin value was counted.

### Ethics

The study protocol was approved by the CHS Ethics Board.

### Statistical analysis

Data were analyzed with SPSS, version 24 (IBM Ltd., Armonk, NY, USA). Categorical variables are described as frequency and percentage, and differences between groups were analyzed with Fisher’s exact test or chi-square test, as appropriate. Continuous variables are described as mean and standard deviation, and differences between groups were analyzed by Student t-test or analysis of variance (ANOVA), as appropriate. A p value < 0.05 was considered significant. Power was defined as β = 0.80.

## Results

The characteristics of the study population are summarized in Table 1. A total of 71,601 infants were included in March 2005 (pre-program baseline; initiation of intervention), 99,036 in March 2014 (end of the intervention), and 107,130 in December 2019 (5 years after end of the intervention). There was no difference in patient characteristics within the target subpopulations between the initiation and end of the intervention program.

**Table 1:** Characteristics of eligible 9–18-month-old population at initiation (2005) and end (2014) of the intervention and 5 years later (2019)

Population	Year		
	2005	2014	2019
All	71,601	99,036	107,130
Gender			
Male, n (%)	36,458	50,739	55,224
	(50.9%)	(51.3%)	(51.5%)
Female, n	35,143	48,297	51,899
Ethnic background			
Jewish, n (%)	43,221	69,146	74,778
	(60.4%)	(69.8%)	(69.8%)
Non-Jewish, n(%)	28,380	29,890	31,709
	(39.6%)	(30.2%)	(29.6%)
Socioeconomic class			
Low, n (%)	37,999	46,864	48,025
	(54.2%)	(48.3%)	(44.8%)
Intermediate, n(%)	23,032	33,892	38,724
	(32.9%)	(34.8%)	(36.1%)
High, n (%)	9,031	16,754	19,320
	(12.9%)	(17.2%)	(18.0%)

Table 2 presents the hemoglobin-testing performance rates for all eligible infants in the sampled populations. Overall, the performance rate was significantly lower at the initiation (in 2005) than at the end (in 2014) of the intervention (54.7% vs 87.5%). There was no significant difference in performance rate between the end of the intervention and 5 years later, in 2019. Analysis by clinical parameters showed that the performance rate increased similarly in males and females to program end (2014). There was no difference between the two ethnic groups, although in 2014, performance was slightly lower in the Jewish subpopulation. A nearly identical performance rate was reached in the various SEC subpopulations by 2014. For all subpopulations, almost no change in performance rate was found from 2014 to 2019.

**Table 2:** Hemoglobin-testing performance rate in eligible 9–18-month-old population at initiation (2005) and end (2014) of the intervention and 5 years later (2019)

Population	Hemoglobin performance/ rate	Hemoglobin performance rate	Change	P value	Hemoglobin performance rate
	2005	2014	2005–2014	2005–2014	2019
All, n (%)	39,190	86,681	60.00%	< 0.01	94,247
	(54.70%)	(87.50%)			(88.0%)
Gender					
Male, n(%)	20,236	44,590	58.40%	< 0.001	48,810
	(55.50%)	(87.90%)			(88.4%)
Female, n(%)	18,954	42,091	61.80%	< 0.001	45,432
	(53.90%)	(87.20%)			(87.5%)
Ethnic background					
Jewish, n(%)	22,873	59,686	63.10%	< 0.001	65,400
	(52.90%)	(86.30%)			(87.5%)
Non-Jewish	16,317	26,995	57.00%	< 0.001	28,661
	(57.50%)	(90.30%)			(90.4%)
Socioeconomic class					
Low	21,973	41,605	53.60%	< 0.001	42,497
	(57.80%)	(88.80%)			(88.5%)
Intermediate	11,190	29,941	81.70%	< 0.001	34,296
	(48.60%)	(88.30%)			(88.6%)
High	5,329	14,676	48.50%	< 0.001	17,049
	(59.00%)	(87.60%)			(88.3%)

**Fig. 1:**
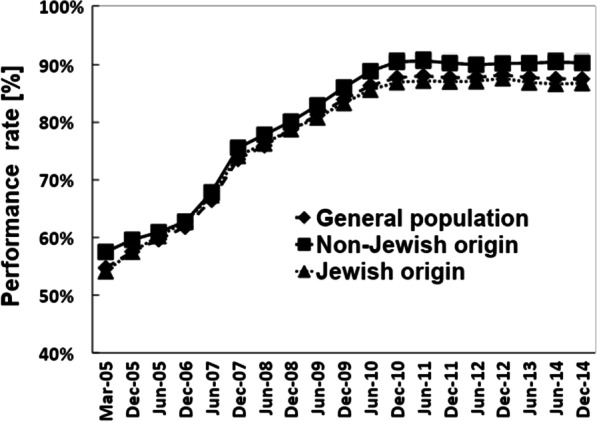
Performance rate of hemoglobin testing in infants aged 9–18 months, 2005–2014, by half-year intervals

Figure 1 presents the pattern of hemoglobin-testing performance rate in the eligible populations in consecutive half-year intervals during the intervention program (2005–2014). Overall, the curve shows a monotonic increase that begins as moderately steep to June 2007, continuing to December 2010, when it plateaus. The curve indicates a consistently higher performance rate in the non-Jewish than the Jewish subpopulation.

Table 3 presents the prevalence rate of IA over time. Overall, the prevalence rate was 7.8% in the respective infants in 2005, and nearly 3.4% in 2014, for a significant 56.4% decrease, with a further decline at 5 years after the intervention ended. By clinical parameters, in 2005, the prevalence rate of IA was higher in males than females and decreased significantly in both genders in 2014. There was a further decline 5 years after the intervention ended. IA remained slightly higher in boys throughout. The prevalence rate was significantly higher in the non-Jewish than the Jewish subpopulation up to 2014 when it equalized at approximately 3.5% in both groups. There was a further decline to below 3% 5 years after the intervention ended. All three SEC subpopulations demonstrated a decreased prevalence rate of IA over time, reaching similar values at the end of intervention (2014), which then declined even further to below 3% after 5 years.

**Table 3:** Prevalence rate of infantile anemia in 9–18-month old children tested for hemoglobin at initiation (2005) and end (2014) of the intervention and 5 years later (2019)

Population	Infantile anemia prevalence rate	Infantile anemia prevalence rate	Prevalence rate change (%)	P value	Infantile anemia prevalence rate
	2005	2014			2019
All, n(%)	3,258	3,135	− 56.40%	< 0.001	2,570
	(7.80%)	(3.40%)			(2.7%)
Gender					
Male, n(%)	1,868	1,801	− 55.80%	< 0.001	1,505
	(8.60%)	(3.80%)			(3.1%)
Female, n(%)	1,390	1,334	− 56.50%	< 0.001	1,065
	(6.90%)	(3.00%)			(2.3%)
Ethnic background					
Jewish, n(%)	1,322	2,150	− 35.80%	< 0.001	1,826
	(5.30%)	(3.40%)			(2.8%)
Non-Jewish, n(%)	1,936	985	− 68.70%	< 0.001	728
	(11.50%)	(3.60%)			(2.5%)
Socioeconomic class					
Low, n(%)	2,246	1,569	− 62.20%	< 0.001	1,144
	(9.80%)	(3.7%)			(2.7%)
Intermediate, n(%)	690	1,055	− 41.10%	< 0.001	927
	(5.60%)	(3.30%)			(2.7%)
High, n(%)	283	480	− 36.20%	< 0.001	474
	(4.70%)	(3.00%)			(2.8%)

**Fig. 2:**
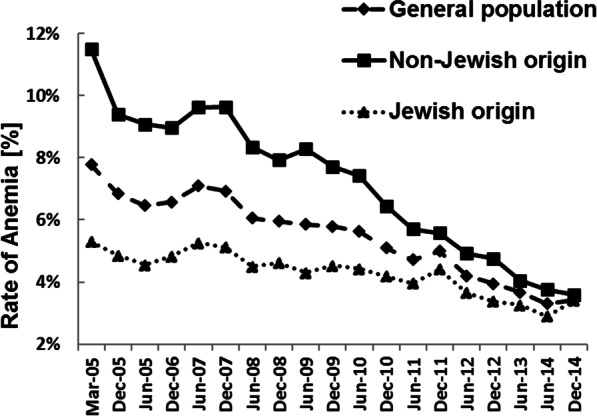
Prevalence rate of anemia in infants aged 9–18 months, 2005–2014, by half-year intervals

Figure 2 presents the pattern of change in the IA prevalence rate in the eligible populations in consecutive half-year intervals during the intervention program (2005–2014). There was a significant and consistent decrease (though fluctuate) in the general population and all subpopulations. The non-Jewish subpopulation had consistently higher prevalence rates of IA than the Jewish subpopulation, but the gap nearly disappeared towards December 2014, almost 10 years after the intervention program was introduced.

The overall prevalence of IA decreased by half (50%) from baseline within 7.25 years (March 2005 to June 2012). In the non-Jewish subpopulation, in which IA prevalence was considerably high, it decreased by half (50%) from baseline within 5.75 years (March 2005 to December 2010), and in the Jewish subpopulation, it decreased by half from baseline within 9.25 years (March 2005–June 2014).

In all subpopulations, there was a further decline 5 years after the intervention ended, in 2019.

## Discussion

### Statement of principal findings

Our study evaluated the progress and yield of an intervention program designed to reduce the prevalence rate of IA. As hemoglobinopathies were excluded, IA served as a proxy for IIDA.

Compared to the Jewish subpopulation, the non-Jewish subpopulation showed a higher rate of performance of hemoglobin testing before initiation of the intervention, but also a considerably higher prevalence rate of IA. There was no difference in performance rate by SEC, although the low-SEC subpopulation had the highest prevalence rate of IA and the high-SEC subpopulation had the lowest prevalence rate.

The pattern of the hemoglobin-testing performance rate over the 10-year intervention was characterized by an S-shaped increase over time, eventually reaching a plateau within 5 years of initiation. Thereafter it remained nearly unchanged to the end of the intervention. The pattern was very similar for both ethnic groups, in which the plateau was reached at 85%-90% of maximum performance (Fig. 1).

The prevalence rate of IA decreased consistently until 2014, and the improvement continued even 5 years after the program ended. This finding suggests that by 2019, hemoglobin testing in children and treatment of IA had become routine practice. In the minority non-Jewish and the low-SEC subpopulations, both of which may be considered weak populations, the prevalence of IA was high at initiation of the intervention and decreased considerably thereafter. By the end of the intervention, ethnicity was no longer a significant factor associated with the prevalence of IA.

### Study strengths

The strength of the study is the large number of consecutive samples of eligible infants over a 10-year period (2005–2014), with an estimated 500,000 individuals aged 9–18 months. All data were derived from electronic medical record documentation, considered a legitimate research source [15]. Hence, we were able to evaluate the yield of the program over time to its termination and ultimately 5 years after.

### Study limitations

In the absence of a control group, we cannot rule out the possibility that several factors other than the targeted intervention program may have contributed to changes in the IA prevalence rate over the 10-year study period. These include food habits, economic conditions, and health services. However, the different patterns by time of improvement in performance rate and the improvement in IA prevalence rate suggest that the main underlying determinant was the intervention program.

### Interpretation within the context of the wider literature

Previous programs aimed at the monitoring and treatment of IIDA have yielded various degrees of success [16–23]. Gill et al. [16] and Morley et al. [18] randomized healthy infants aged 6 and 9 months, respectively, to feeding with iron-fortified formula, non-iron fortified formula, or cow's milk. Both studies reported a significant increase in hemoglobin and ferritin levels. Accordingly, Lozoff et al. [21] observed a significant increase in the same two parameters in 1657 infants treated with multiple interventions with varying iron concentrations. However, in a placebo-controlled study of 70 infants aged 4–9 months, Domellöf et al. [20] reported a significant increase in serum ferritin but not in hemoglobin, and both Makrides et al. [21] and Geltman et al. [22] noted no significant difference in hemoglobin or serum ferritin levels between infants fed a high-iron weaning diet and controls or between infants fed standard-dose iron-fortified or non-iron fortified oral multivitamins. The differences in results among the studies might be attributable to their different designs. Furthermore, various cut-off points were used to define anemia, ranging from 105 g/L to 110 g/L [10, 14, 23, 24].

Both the present study and our previous one [14] found that the rate of IA among infants in Israel was higher than in other developed countries [3–5]. The reason is unknown [3, 25, 26]. This study proves that the high prevalence rate is manageable with the proper intervention.

Although some studies reported an improvement in hematologic values following intervention, two recent systematic reviews showed no evidence of a long-term effect on clinical parameters [5, 27]. These reviews join the plethora of longitudinal studies consistently indicating that children who are anemic in infancy continue to have poor cognition, auditory and visual dysfunctions, low scholastic achievements, and behavioral problems into middle childhood. Short-term iron treatments failed to produce benefits in development, and even correction of the IDA did not alleviate these long-term problems [21, 28–32].

These findings raise questions regarding the current practice of initiating hemoglobin testing at the age of 9 months. Although the American Academy of Pediatrics advocates screening of all infants aged 9 to 12 months [33], the U.S. Preventive Task Force (USPSTF) statements of 2006 and 2015 found the evidence insufficient to support a general recommendation for routine screening for IDA in asymptomatic children in the 6- to 12-month age group. The USPSTF suggests that iron supplements be routinely used only in cases of increased risk of IDA [26, 34, 35].

Our population differs from the population referred to in the USPSTF statements in terms of two important factors: (1) The rate of infantile anemia was high relative to other developed countries, which may make the effect of intervention more pronounced. (2) Relatively high proportions of the non-Jewish and low socioeconomic subpopulations were more affected by the intervention. These patient groups were not included in the USPSTF population base [34, 35].

Furthermore, although treating existent anemia may not affect the clinical outcome, effective early detection and treatment may avoid the adverse effects of IDA on school performance, behavioral alterations, and growth. It may also reduce the economic burden of IDA on the country’s healthcare system.

### Implications for policy

A high rate of performance of hemoglobin testing does not guarantee a low prevalence rate of IA. However, hemoglobin testing is a preliminary condition to diagnosis. The calculation of the prevalence rate of IA in this study was limited to patients who were tested and was undetermined in those untested.

We showed that the intervention program benefits weak subpopulations (minorities, low SEC) no less than strong subpopulations.

### Practice and research

This study provides important insights into the pattern of change in the rates of hemoglobin testing and prevalence of IA.

Retrospective research based on registries or existing databases is an acceptable means to evaluate health proceedings or patterns, albeit with awareness of the design limitations. The use of real-world evidence has long been debated and was recently categorized by Collins et al. [36]. We believe our study, by using a large database from real life while considering possible confounders, provides sufficient and valid evidence of the importance of the quality indicators of performance rate and prevalence rate for assessing the efficacy and yield of intervention programs for health improvement.

## Conclusions

Our findings strongly suggest that the implementation of a well-designed intervention program accompanied by monitoring measures, combined with an organizational focus and treatment guidelines, effectively reduced the prevalence rate of IA in the target population. The main concerns directed at intervention programs are whether they maintain an impact over time and if they benefit all subpopulations equally. We proved that the benefit persisted even 5 years after the intervention ended and that the yield of the intervention was equal for the whole population and across the subpopulations evaluated.

Our study strongly suggests that an intervention program that provides continuous feedback to clinicians is an excellent tool for improving outcome of a common clinical problem such as IA. Furthermore, that the improvement in our study continued even 5 years after the program ended suggests that the process of performing blood counts, initiating early preventive treatment, and treating IA accordingly had become common practice.

We recommended to the Israel Ministry of Health to adopt our intervention program countrywide. We challenge other countries, especially those in which the population or subpopulations have a high prevalence of IA, to consider similar programs.

## Data Availability

No data sharing is available.

## Abbreviations

CHS: Clalit Health Services
IDA: Iron-deficiency anemia
IA: Infantile anemia
IIDA: Infantile iron-deficiency anemia
SEC: Socioeconomic class
